# Patients’ perspective on the burden of migraine in Europe: a cross-sectional analysis of survey data in France, Germany, Italy, Spain, and the United Kingdom

**DOI:** 10.1186/s10194-018-0907-6

**Published:** 2018-09-10

**Authors:** Pamela Vo, Juanzhi Fang, Aikaterini Bilitou, Annik K. Laflamme, Shaloo Gupta

**Affiliations:** 1 Novartis Pharma AG, Fabrikstr. 12, CH-4002 Basel, Switzerland; 2 Novartis Pharmaceuticals Corporation, One Health Plaza, East Hanover, NJ 07936-1080 USA; 3 Novartis Global Services Centre, Patient Access Services, Dublin, Ireland; 4 Kantar Health, New York, NY 10010 USA

**Keywords:** Activity impairment, Burden, Healthcare resource use, Health-related quality of life, Migraine, Work impairment

## Abstract

**Background:**

Migraine is a distinct neurological disease that imposes a significant burden on patients, society, and the healthcare system. This study aimed to characterize the incremental burden of migraine in individuals who suffer from ≥4 monthly headache days (MHDs) by examining health-related quality of life (HRQoL), impairments to work productivity and daily activities, and healthcare resource utilization (HRU) in the EU5 (France, Germany, Italy, Spain, United Kingdom).

**Methods:**

This retrospective cross-sectional study used data from the 2016 National Health and Wellness Survey (NHWS; *N* = 80,600). Short-Form 36-Item Health Survey, version 2 (SF-36v2) physical and mental component summary scores (PCS and MCS), Short-form-6D (SF-6D), and EuroQoL (EQ-5D), impairments to work productivity and daily activities (Work Productivity and Activity Impairment Questionnaire (WPAI), and HRU were compared between migraine respondents suffering from ≥4 MHDs (*n* = 218) and non-migraine controls (*n* = 218) by propensity score matching using sociodemographic characteristics. Chi-square, T-tests, and Mann-Whitney tests were performed to determine significant differences between the groups after propensity score matching.

**Results:**

HRQoL was lower in migraine individuals suffering from ≥4 MHDs compared with non-migraine controls, with reduced SF-36v2 PCS (46.00 vs 50.51) and MCS (37.69 vs 44.82), SF-6D health state utility score (0.62 vs 0.71), and EQ-5D score (0.68 vs 0.81) (for all, *p* < 0.001). Respondents with migraine suffering from ≥4 MHDs also reported higher levels of absenteeism from work (14.43% vs 9.46%; *p* = 0.001), presenteeism (35.52% vs 20.97%), overall work impairment (38.70% vs 23.27%), and activity impairment (44.17% vs 27.75%) than non-migraine controls (for all, *p* < 0.001). Additionally, HRU was significantly higher for individuals with ≥4 MHDs compared to their matched controls. Consistently, migraine subgroups (4–7 MHDs, 8–14 MHDs and CM) had lower HRQoL, greater overall work and activity impairment, and higher HRU compared to non-migraine controls.

**Conclusions:**

Migraine of ≥4 MHDs was associated with poorer HRQoL, greater work productivity loss, and higher HRU compared with non-migraine controls. The findings of the study suggest that an unmet need exists among individuals suffering from ≥4 MHDs in the EU5 suggesting the need for effective prophylactic treatments to lessen the humanistic and economic burden of migraine.

## Background

Migraine is a distinct neurological disease, associated with recurrent and often debilitating headaches of moderate to severe intensity and accompanied by neurological symptoms (sensory and dysautonomic symptoms including nausea, vomiting, photophobia, or phonophobia) that exact a personal, economic, and societal burden on a global scale [1]. Migraine has been categorized into 2 major types: migraine with aura and migraine without aura. The former accounts for around 30% of the patients and involves transient visual, sensory, and aphasic or motor disturbances that occur before or during migraine attacks [2]. A single migraine attack typically disrupts patient’s life and can consist of premonitory (≤48 h), aura (5–60 min), headache (4–72 h), and resolution/postdrome (≤48 h) phases [3]. Migraine generally starts during puberty and is most prevalent between 30 and 49 years of age [4]. Migraine affects approximately > 10% of the adult population globally [5], is 2–3 times more common in women than men, and tends to run in families and has a genetic trend [6].

Migraine can be immensely disabling [7] and impacts a patient’s functional ability and health-related quality of life (HRQoL) during, immediately after, and between migraine episodes [8].

Migraine was the sixth leading cause of disability-adjusted life years (DALYs) worldwide for the age group 25 to 39 years in the 2015 Global Burden of Disease (GBD) study [9]. The GBD 2016 study reported migraine as the first leading cause of years lived with disability (YLDs) worldwide in both males and females for the age group 15 to 49 years, demonstrating that the burden is higher in the groups of prime productivity [10]. In fact, migraine-attributed YLDs were much higher in comparison to other neurological diseases such as epilepsy (ranked 29th) and Alzheimer disease (ranked 26th) [11].

The burden associated with migraine is underestimated even in developed countries despite its high prevalence and severity [12]. Although the prevalence of migraine in individuals suffering from ≥4 monthly headache days (MHDs) is lower when compared to <3MHDs [13], the burden is higher [14]. Studies based on sociodemographic [15] and general health characteristics of migraine [16, 17], HRQoL [18, 19], work productivity loss and activity impairment (WPAI) [17], and healthcare resource utilization (HRU) [17] have been conducted before. Furthermore, a number of studies across multiple countries have studied the impact of chronic and episodic migraine on HRQoL, WPAI, and HRU [7, 18, 20–23]. However, there is paucity of data on HRQoL, WPAI, and HRU for the entire spectrum of migraine in the EU5 (France, Germany, Italy, Spain, and the United Kingdom) especially in the population with migraine who suffer from ≥4MHDs and may be eligible for prophylactic treatment [13, 24–29].

Therefore, the primary objective of this study was to characterize the incremental burden of migraine in those experiencing ≥4 MHDs from patients’ perspective in terms of HRQoL, work and activity impairment, and HRU compared with non-migraine controls among the EU5. The secondary objective was to characterize the burden of migraine from the perspective of migraine patients experiencing ≥4 MHDs from the EU5 by frequency of migraine (eg, 4–7, 8–14, and ≥15 MHDs) compared with non-migraine controls.

## Methods

### Sample

The sample for this retrospective, cross-sectional study was taken from the 2016 National Health and Wellness Survey (NHWS; *N* = 80,600) from adults in the EU5. All respondents were aged 18 years or older, consented to participate in the survey, and could read and write the primary language of the country at the time of the survey.

Respondents to this NHWS are members of MySurvey.com or its partners, which are opt-in survey panels, who were recruited through opt-in e-mail, co-registration with MySurvey.com partners, eNewsletter campaigns, banner placements, and both internal and external affiliate networks. All potential panelists must register with the panel through a unique e-mail address and password and complete an in-depth demographic registration profile. In countries where Internet penetration among the elderly was not considered sufficient to provide an adequate sample of the elderly population (Spain and Italy), telephone recruitment using quota sampling (age and gender) was used to supplement online recruitment, and those without access to the internet were invited to complete the survey using a computer in a private center. The protocol and questionnaire for the NHWS were reviewed for exemption by Pearl Institutional Review Board (IRB) and determined to be exempt from IRB review for the periods the data used in the current study; all respondents provided informed consent.

**Fig. 1:**
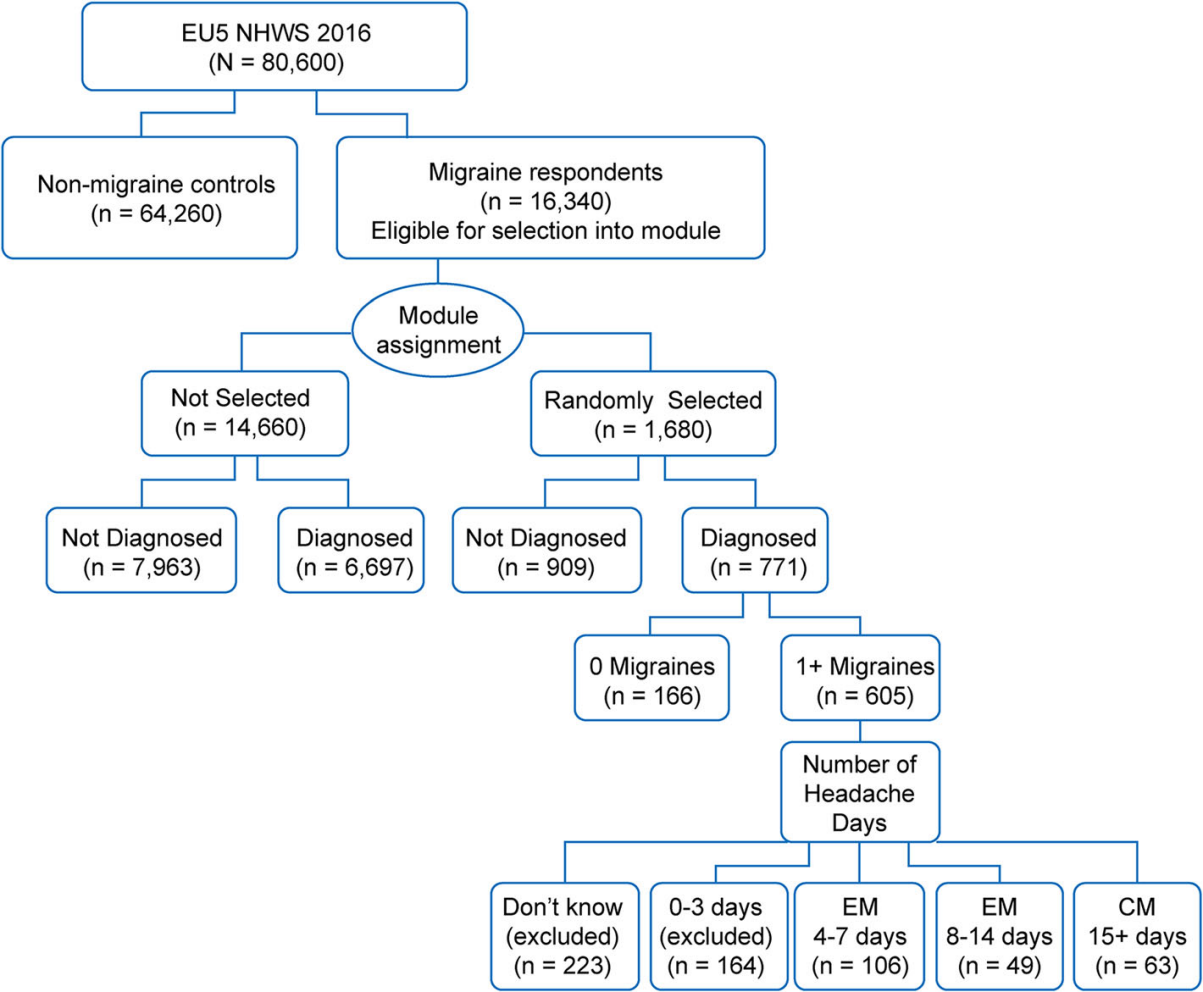
Selection of study population CM, chronic migraine; EM: episodic migraine; EU5, France, Germany, Italy, Spain, and the United Kingdom; NHWS, National Health Wellness Survey; n, the total number of respondents across the EU5.

Of the 16,340 survey respondents who reported experiencing migraine in the past 12 months, a randomly selected subsample of 1680 respondents (10%) completed the migraine module with additional questions on migraine characteristics and of these, 771 respondents reported a physician-diagnosed migraine. Such random subsampling enabled inclusion of respondents with different conditions to provide detailed information while limiting the average interview length and respondent’s burden. As the objective was to evaluate the burden of migraine in respondents with ≥4 MHDs, respondents who did not experience migraines in the past month or did not provide a frequency of MHDs or reported rare migraine (≤3 MHDs) were excluded from the study (*n* = 553) and 218 respondents were included for the study (Fig. 1).

The study sample (respondents who self-reported a physician-diagnosis of migraine) who completed the migraine module and indicated that they experienced migraines of at least 4 MHDs were matched by propensity scores to those without migraines (controls) using sociodemographic characteristics (see below). Furthermore, respondents were categorized according to the frequency of migraines (MHDs): non-migraine controls, people with migraine of 4 to 7 MHDs (4–7 EM), 8 to 14 MHDs (8–14 EM), and 15 or more MHDs (CM) [3].

### Measures

#### Sociodemographic characteristics

The demographic characteristics collected included country of residence (i.e., France, Germany, Italy, Spain, and the United Kingdom), age in years, gender (male or female), employment status (yes vs no), annual household income (below median vs above median vs decline to answer), marital status (married or living with partner vs not), and level of education (completed university education vs not).

#### General health characteristics

Body mass index (BMI) was calculated from reported height and weight and reported as underweight (< 18.5 kg/m^2^), normal weight (18.5 to < 25.0 kg/m^2^), overweight (25 to < 30.0 kg/m^2^), obese (30.0 kg/m^2^ and above), or decline to answer. Other general characteristics collected were cigarette smoking (current vs former vs never); alcohol use (yes vs no); vigorously exercised in past 30 days (yes vs no); and the Charlson Comorbidity Index (CCI) [30]. CCI weights the presence of various conditions [eg, diabetes, liver disease, connective tissue disease, chronic pulmonary disease, metastatic tumor, moderate/severe renal disease, peripheral vascular disease, myocardial infarction, congestive heart failure, diabetes with end organ damage, leukemia, dementia, and human immunodeficiency virus infection/acquired immune deficiency syndrome (HIV/AIDS)] and sums the result. The greater the total index score, the greater is the comorbid burden on the patient.

#### Health-related quality of life

##### SF-36v2

The 2016 NHWS included the 4-week recall period of the revised Medical Outcomes Study 36-Item Short-Form Survey Instrument (SF-36v2), which is a multipurpose, generic health status instrument that consists of 36 questions [31]. Two SF-36 summary scores were calculated, physical component summary (PCS) and mental component summary (MCS) scores, with higher scores indicating better HRQoL. In addition to generating profile and summary PCS and MCS scores, the SF-36v2 can also be used to generate health state utilities, similar to those derived from the EuroQoL EQ-5D (EQ-5D). This is achieved through the application of the Short-Form Six-Dimension (SF-6D), which takes 6 items from the survey.

The SF-6D is a preference-based single index measure for health using UK general population values [32]. The SF-6D index has interval scoring properties and yields summary scores on a theoretical scale of 0 to 1. Higher scores indicate better health status. The EQ-5D Index score is a preference-based measure of health on a theoretical scale of 0 to 1, in which 1 represents full health and 0 being death. It is derived from responses to the 5-level EQ-5D version (EQ-5D-5 L), a widely used survey instrument that measures health in 5 dimensions, which was included in the questionnaire for this study [33].

#### Work productivity and activity impairment

Loss of productivity and activity impairment were assessed using the General Health version of the Work Productivity and Activity Impairment (WPAI-GH) questionnaire [34], a 6-item validated instrument that consists of 4 metrics: absenteeism (the percentage of work time missed because of one’s health in the past 7 days), presenteeism (the percentage of impairment experienced while at work in the past 7 days because of one’s health), overall work productivity loss (overall work impairment measured by combining absenteeism and presenteeism to determine the total percentage of missed time), and activity impairment (the percentage of impairment in daily activities because of one’s health in the past 7 days). Only respondents who reported being full-time, part-time, or self-employed provided the data for absenteeism, presenteeism, and overall work impairment. All respondents completed the activity impairment questionnaire.

#### Healthcare resource utilization

HRU was defined by visits to different medical providers or healthcare system (i.e., Emergency department [ED] or hospital) 6 months before survey participation due to any medical conditions, not only migraine specific. Several types of healthcare provider (HCP) visits were summarized and analyzed as the presence versus absence of a visit in the prior 6 months as well as the number of visits during that time. The proportion of respondents using healthcare resources was summarized. The HRU of respondents included HCP visits overall, primary care provider visits, neurologist visits, psychiatrist visits, ED/urgent care visits, and hospitalization in the past 6 months.

### Matching

As the objective of the study was to estimate the incremental burden associated with migraine, the propensity score of respondents with migraine was compared with that of those without migraine (controls) using demographic and comorbidities data. This procedure was conducted separately within each country and for those with 4–7 EM, 8–14 EM, and CM to limit the risk that respondents differ from controls on matching characteristics within the smaller migraine subgroups.

Logistic regressions including sociodemographic and health variables (age, sex, marital status, income, education, smoking status, alcohol use, exercise behavior, BMI category, and CCI [estimate of comorbidity burden]) were conducted. Using a greedy matching algorithm, respondents’ regression-estimated probabilities were used to match each case to a single control with no reuse of controls. Matching was constrained so that each respondent with migraine was matched to a non-migraine control from the same country.

### Data analysis plan

All data management and analyses were performed in SPSS 23.0, and SAS 9.4. The sample was characterized by the variables listed in the variables section using descriptive statistics, including frequencies and percentages for categorical variables, and means, standard deviations for continuous variables.

Differences between respondents diagnosed with migraine versus non-migraine controls were examined. For categorical variables, chi-square tests were used to determine significant differences, whereas t-tests or the Mann-Whitney tests, where appropriate, were used for continuous variables.

## Results

Among respondents who reported a physician-diagnosed migraine, suffer from ≥4 MHDs, and completed the migraine module (*N* = 218), 67 (30.7%) respondents were from the United Kingdom, 59 (27.1%) from Germany, 39 (17.9%) from France, 31 (14.2%) from Italy, and 22 (10.1%) respondents from Spain. The demographic characteristics for respondents diagnosed with migraine and pre-matched and post-matched non-migraine controls are represented in Table 1.

**Table 1:** Demographic characteristics for respondents diagnosed with migraine and pre-matched and post-matched non-migraine controls

		Diagnosed with migraine, *N* = 218	Pre-matched non-migraine controls, *N* = 64,260	Post-matched non-migraine controls, *N* = 218
Gender, n (%)	Male	45 (20.64)	31,275 (48.7)***	44 (20.18)
	Female	173 (79.36)	32,985 (51.3)	174 (79.82)
Country, n (%)	France	39 (17.89)	15,524 (24.2)	39 (17.89)
	Germany	59 (27.06)	16,635 (25.9)	59 (27.06)
	United Kingdom	67 (30.73)	15,616 (24.3)	67 (30.73)
	Italy	31 (14.22)	8868 (13.8)	31 (14.22)
	Spain	22 (10.09)	7617 (11.9)	22 (10.09)
Marital status, n (%)	Married or living with partner	131 (60.09)	39,943 (62.2)	131 (60.09)
Household income, n (%)	Low	63 (28.90)	16,219 (25.2)	57 (26.15)
	Medium	100 (45.87)	28,743 (44.7)	108 (49.54)
	High	36 (16.51)	12,462 (19.4)	38 (17.43)
	Decline to answer	19 (8.72)	6836 (10.6)	15 (6.88)
Level of education, n (%)	Completed university education	87 (39.91)	22,958 (35.7)	90 (41.28)
Employment status, n (%)	Yes	136 (62.39)	34,640 (53.9)*	131 (60.09)
	No	82 (37.61)	29,620 (46.1)	87 (39.91)
BMI, n (%)	Underweight (< 18.5 kg/m^2^)	4 (1.83)	1947 (3.0)	4 (1.83)
	Normal weight (18.5 to < 25.0 kg/m^2^)	87 (39.91)	26,935 (41.9)	83 (38.07)
	Overweight (25 to < 30.0 kg/m^2^)	66 (30.28)	21,002 (32.7)	75 (34.40)
	Obese (30.0 kg/m^2^ and above)	48 (22.02)	11,135 (17.3)	48 (22.02)
	Decline to answer	13 (5.96)	3241 (5.0)	8 (3.67)
Smoking status, n (%)	Current	66 (30.28)	14,886 (23.2)*	67 (30.73)
	Former	66 (30.28)	20,420 (31.8)	70 (32.11)
	Never	86 (39.45)	28,954 (45.1)	81 (37.16)
Use of alcohol, n (%)	Yes	153 (70.18)	49,749 (77.4) *	148 (67.89)
	No	65 (29.82)	14,511 (22.6)	70 (32.11)
Vigorous exercise in past 30 days, n (%)	Yes	126 (57.80)	40,342 (62.8)	121 (55.50)
	No	92 (42.20)	23,918 (37.2)	97 (44.50)
Age [mean ± SD]	Years	43.25 ± 13.48	16.57 ± 49.79***	44.73 ± 16.09
CCI [mean ± SD]		0.53 ± 1.04	0.82 ± 0.33***	0.44 ± 1.01

Note: Chi-square tests were used for categorical variables, whereas t-tests were used for continuous variables

*p* -values for pre-matched non-migraine controls vs migraine group: *p < 0.05, *** *p* ≤ 0.001

*BMI*, Body mass index; *CCI*: Charlson Comorbidity Index; *SD*, standard deviation

### Comparison of respondents with migraine and its subgroups with non-migraine controls

#### Health-related quality of life

**Fig. 2:**
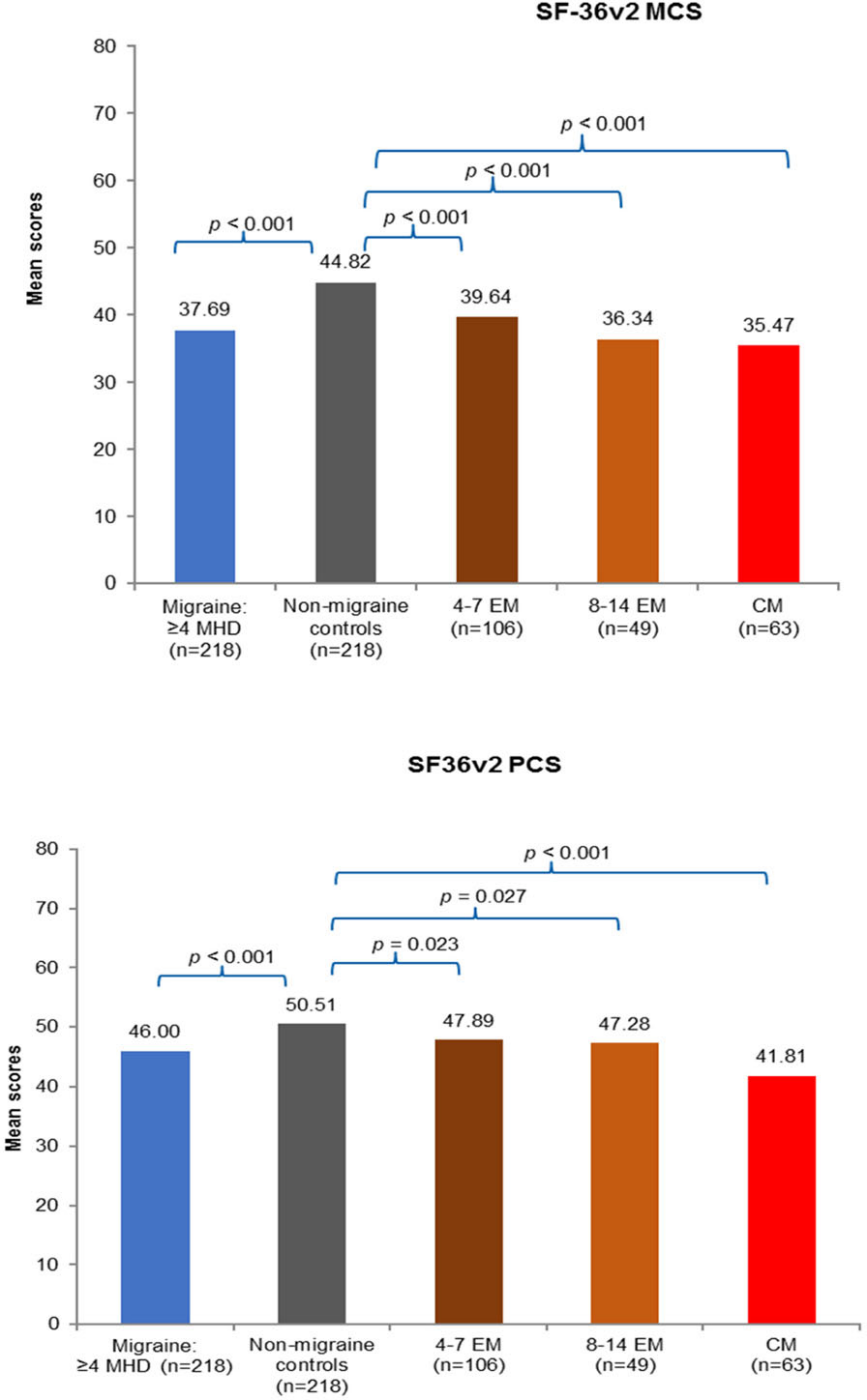
MCS and PCS scores of SF-36v2 of migraine subgroups vs propensity score matched non-migraine controls T-tests were used for analysis. CM, chronic migraine; EM, episodic migraine; MCS, mental component summary; MHD, monthly headache day; PCS, physical component summary.

**Fig. 3:**
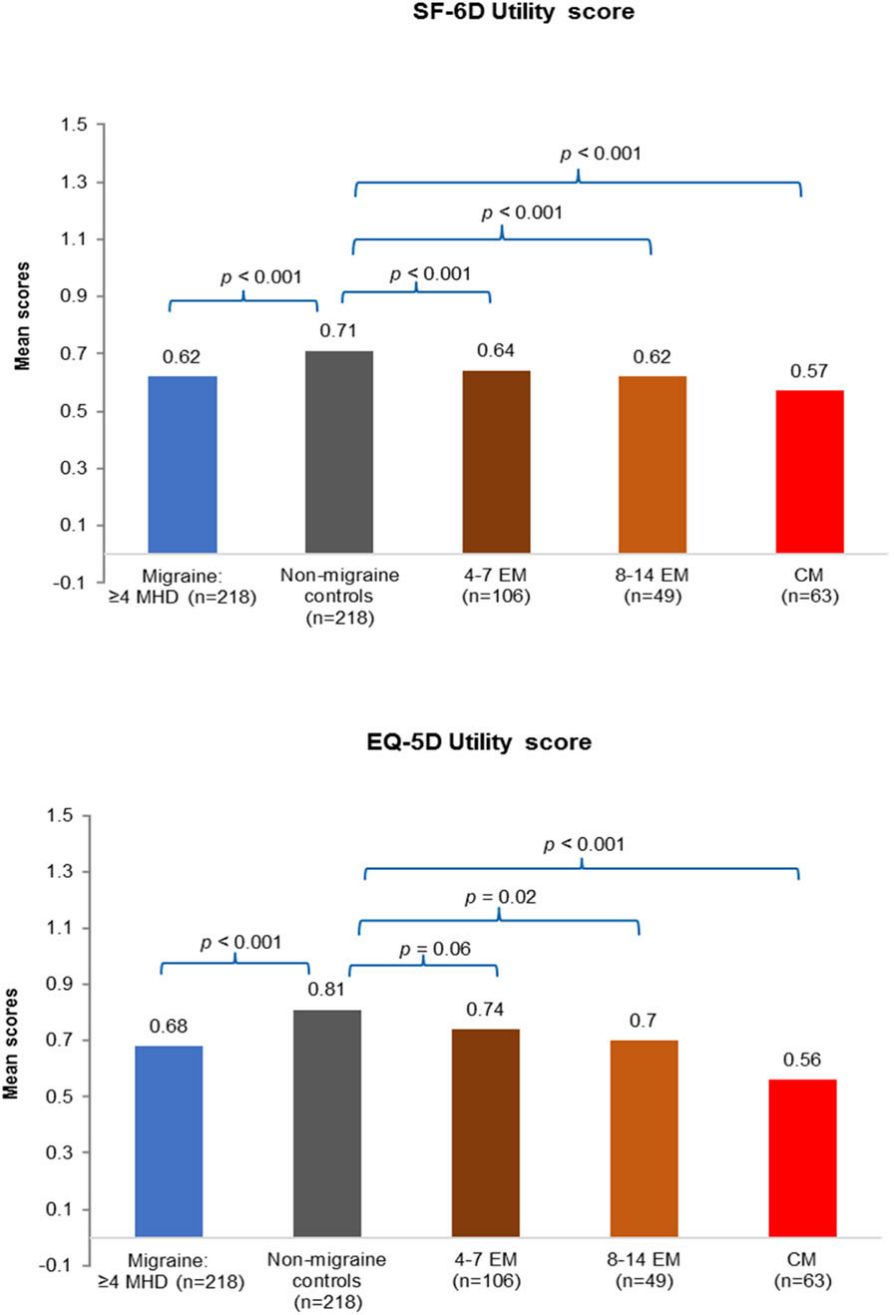
SF-6D health utility score and EQ-5D Index of migraine subgroups vs propensity score matched non-migraine controls T-tests were used for analysis. CM, chronic migraine; EM, episodic migraine; EQ-5D, EuroQol-5D; MHD, monthly headache day; QoL, quality of life.

The results from the propensity score matched analysis demonstrated that individuals with migraine who suffer from ≥4 MHDs reported statistically significant lower HRQoL than non-migraine controls, both mentally and physically, as measured by the SF-36v2. The MCS in those with migraine was significantly lower than that in non-migraine controls (37.7 vs 44.8, respectively; *p* < 0.001; Fig. 2). Furthermore, the PCS score of migraine respondents was significantly lower than that of non-migraine controls (46.0 vs 50.5, respectively; *p* < 0.001; Fig. 2), this represents an incremental difference of 7.1 in MCS and 4.5 in PCS which is greater than the previously reported MCS and PCS mean for a minimally important difference of 3 [35]. Respondents with migraine also reported significantly lower SF-6D (0.62 vs 0.71, *p* < 0.001) and EQ-5D (0.68 vs 0.81, *p* < 0.001) health utility scores than the non-migraine controls (Fig. 3); this represents an incremental difference of 0.09 which is greater than the previously reported mean for a minimally important difference of 0.041 [36]. The significant decrement in PCS and MCS scores was noted across migraine frequency subgroups compared to non-migraine controls suggesting that migraine impacts HRQoL irrespective of frequency (Fig. 2). Furthermore, an increase in the number of MHDs was associated with worse SF-6D utility and EQ-5D health status scores compared to non-migraine controls (Fig. 3).

#### Work productivity impairment and activity impairment

**Fig. 4:**
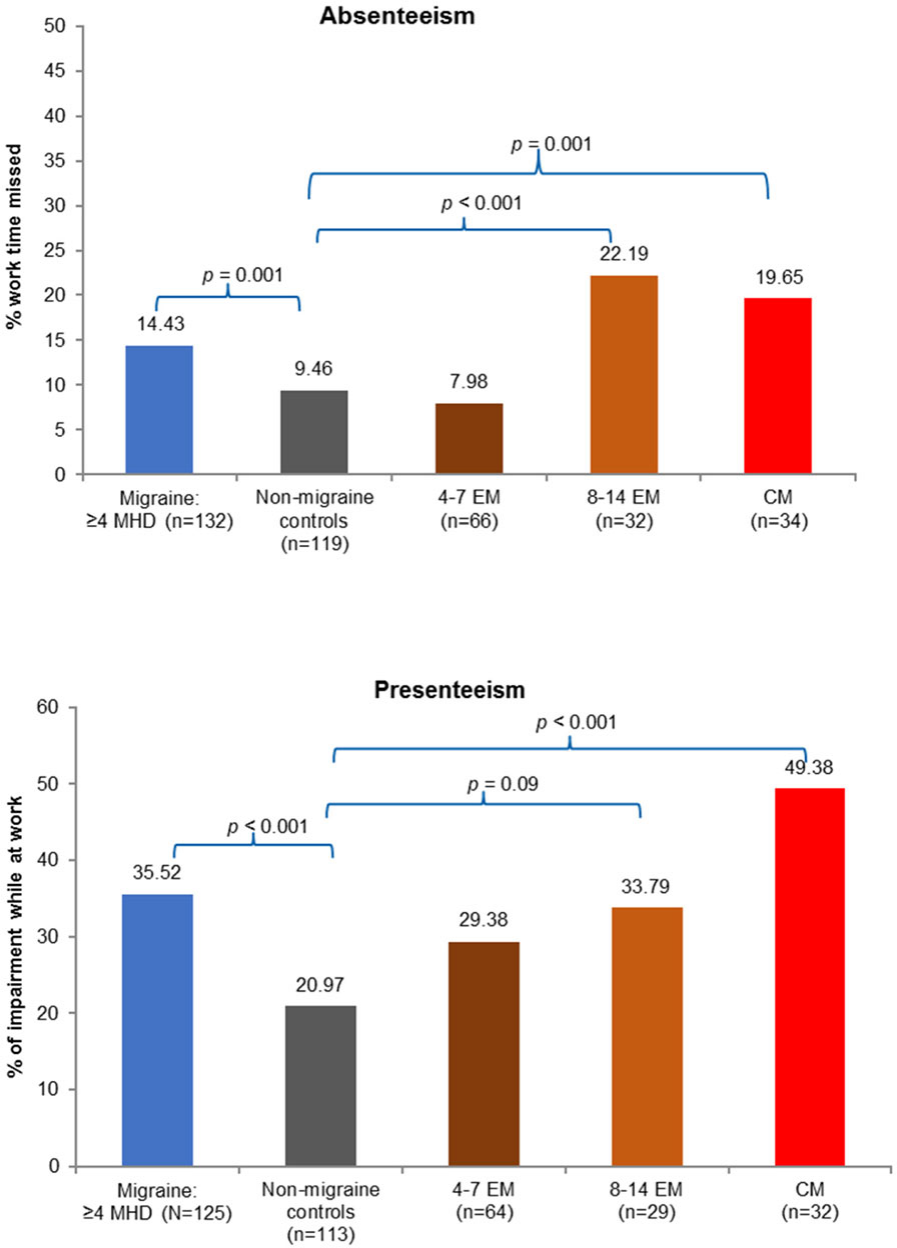
Work productivity loss in migraine subgroups vs propensity score matched non-migraine controls by the WPAI metrics Mann-Whitney tests were used for analysis. CM, chronic migraine; EM, episodic migraine; MHD, monthly headache day; WPAI, work productivity and activity impairment.

**Fig. 5:**
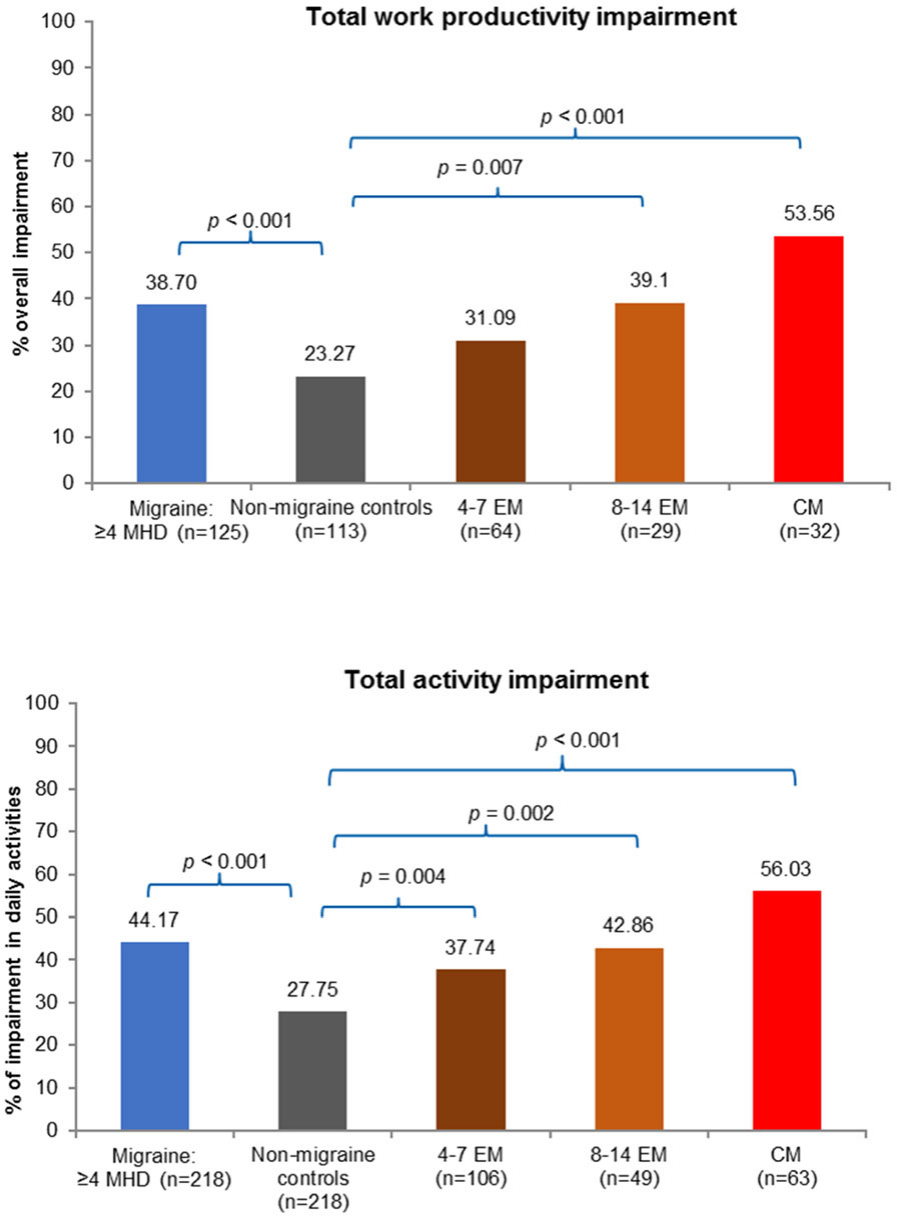
Total work productivity and activity impairment in migraine subgroups vs propensity score matched non-migraine controls Mann-Whitney tests were used for analysis. CM, chronic migraine; EM, episodic migraine; MHD, monthly headache day.

Respondents with migraine when compared with non-migraine controls reported significantly higher absenteeism (14.4% vs 9.5%, respectively; *p* = 0.001; Fig. 4) and presenteeism (35.5% vs 21.0%, respectively; *p* < 0.001; Fig. 4). Higher incremental presenteeism vs non-migraine controls were noted across the migraine sample irrespective of migraine frequency (Fig. 4). Among employed respondents, the total work productivity impairment including both absenteeism, presenteeism, and among all respondents activity impairment was significantly higher in those with migraine than non-migraine controls (38.7% vs 23.3% and 44.2% vs 27.8%, respectively; Fig. 5).

#### Healthcare resource utilization

HRU was significantly higher in the migraine sample compared with non-migraine controls (Table 2). In the past 6 months before completion of questionnaire, the mean number of total HCP visits (8.5 vs 5.1; *p* < 0.001) and ED visits (0.46 vs 0.21; *p* = 0.011) reported by the migraine sample were significantly higher than non-migraine controls. In particular, the mean general/family practitioner visits (3.1 vs 1.7; *p* < 0.001), neurologist visits (0.19 vs 0.05, *p* < 0.001), and psychiatrist visits (0.85 vs 0.15; *p* < 0.001) were significantly higher for the migraine sample when compared with the non-migraine controls. A significantly higher proportion of migraine respondents compared with non-migraine controls had at least one visit to a general/family practitioner (77.1% vs 67.4%; *p* = 0.025), neurologist (13.8% vs 3.7%; *p* < 0.001), and psychiatrist (13.3% vs. 3.2%; *p* < 0.001) in the prior 6 months.

**Table 2:** Healthcare resource utilization in the past 6 months among people with migraine and its subgroups vs propensity score matched non-migraine controls across EU5

HRU	Migraine (n = 218)	Non-migraine controls(*n* = 218)	4–7 EM (*n* = 106)	8–14 EM(*N* = 49)	CM(*N* = 63)
Number of any HCP visits [mean ± SD]	8.48 ± 10.89***	5.13 ± 6.86	7.25 ± 7.29	7.06 ± 7.64	11.65 ± 16.30
Number of general/ family practitioner visits [mean ± SD]	3.08 ± 3.39***	1.67 ± 1.93	3.05 ± 3.38*	2.94 ± 3.42	3.24 ± 3.43*
Number of neurologist visits [mean ± SD]	0.19 ± 0.54***	0.05 ± 0.29	0.20 ± 0.59***	0.16 ± 0.51*	0.19 ± 0.47***
Number of psychiatrist visits [mean ± SD]	0.85 ± 4.48***	0.15 ± 0.86	0.36 ± 1.22**	0.41 ± 1.58*	2.02 ± 7.98***
Number of ED visits [mean ± SD]	0.46 ± 1.20*	0.21 ± 0.79	0.27 ± 1.06	0.61 ± 1.24**	0.67 ± 1.33**
Number of hospitalizations [mean ± SD]	0.18 ± 0.54	0.11 ± 0.53	0.12 ± 0.38	0.16 ± 0.43	0.30 ± 0.78*
Visited any HCP [n, %]	204 (93.58%)	193 (88.53%)	100 (94.3%)	44 (89.8%)	60 (95.2%)
Visited general /family practitioner [n, %]	168 (77.06%)*	147 (67.43%)	83 (78.3%)*	34 (69.4%)	51 (81.0%)*
Visited neurologist [n, %]	30 (13.76%)***	8 (3.67%)	14 (13.2%)***	6 (12.2%)*	10 (15.9%)***
Visited psychiatrist [n, %]	29 (13.30%)***	7 (3.21%)	11 (10.4%)**	5 (10.2%)*	13 (20.6%)***
ED visits [n, %]	45 (20.64%)*	27 (12.39%)	12 (11.3%)	15 (30.6%)**	18 (28.6%)**
Hospitalization [n, %]	28 (12.84%)	16 (7.34%)	11 (10.4%)	7 (14.3%)	10 (15.9%)*

Note: Chi-square tests were used to analyze categorical variables, whereas Mann-Whitney tests were used to analyze continuous variables

*p* -values for non-migraine controls vs any other group: *p < 0.05, **p < 0.01, *** *p* ≤ 0.001

*CM*, chronic migraine; *ED*, emergency department; *EM*, episodic migraine; *HCP*, healthcare provider i.e. any physician; *HRU*, healthcare resource utilization; *SD*, standard deviation

The mean number of hospitalizations in a 6-month period prior to survey was also higher among those with migraine, although marginally significant, compared with non-migraine controls (0.18 vs 0.11; *p* = 0.056). The proportion of respondents who reported at least one ED visit was significantly higher in the migraine group than non-migraine control (20.6% vs. 12.4%; *p* = 0.02) whereas the proportion hospitalized (12.8% vs. 7.3% *p* = 0.056) was higher but marginally significant.

## Discussion

The study used responses from patients of a randomly selected subsample who completed the migraine module (10%), and also reported a physician-diagnosed migraine with ≥4 MHDs (Fig. 1); a patient population that is often deemed eligible for prophylactic treatment in clinical trials and practice. The analysis showed that after propensity score matching of the subgroups based on demographic and health characteristics, those suffering from migraine of at least 4 MHDs had significantly lower HRQoL, increased work and activity impairment, and higher HRU than their non-migraine matched controls. The incremental burden due to migraine was demonstrated in all domains of life across migraine frequency subgroups in the migraine spectrum with ≥4 MHDs (4–7 EM, 8–14 EM, and CM), suggesting that every migraine attack is associated with burden impacting the well-being, productivity, and HRQoL of individuals affected. This study used the self-reported data from the 2016 NHWS to provide evidence on multiple dimensions of HRQoL, WPAI, and HRU. The NHWS is a validated recurrent survey conducted across multiple countries and multiple therapy areas using standardized questionnaires to study disease-associated burden [37–40]. The methodology used ensures representativeness of the general population and is, hence, useful to understand the overall disease burden within a country.

The poorer HRQoL observed in our study in terms of lower physical, mental, and overall health status (utility scores) in those with migraine compared with non-migraine controls is consistent with previous published studies [41, 42]. Similar studies using SF-36 showed migraine to be associated with lower HRQoL in terms of physical functioning, bodily pain, general health perception, vitality, social functioning, emotional role, and mental health in Turkish patients [43] and Malaysian female patients [44]. Furthermore, our results of the SF-6D in those with migraine vs their matched controls showed that migraine is associated with a minimally important decrement in utility.

The Eurolight study which was conducted across multiple countries (Austria, France, Germany, Italy, Lithuania, Luxembourg, Netherlands, Spain, and UK) showed that migraine is associated with personal impact that affects personal, work, housework and social activities in both men and women who suffer from migraine [8] and therefore significant economic burden to the society [45]. .The American Migraine Prevalence and Prevention (AMPP) and other international studies have also shown that migraine impacts all aspects of life and that the burden increases with frequency [7, 46].

The present study showed higher levels of absenteeism (1.5-fold), presenteeism (1.7-fold), work productivity impairment (1.7-fold), and activity impairment (1.6-fold) in those suffering from at least 4 MHDs compared with non-migraine matched controls. This is consistent to previously published studies reporting that migraine can result in substantial loss in useful time and productivity, especially in the form of presenteeism (impaired productivity while at work) [8, 45].

The high burden of migraine is depicted as DALYs and YLDs in the Global Burden Disease study, 2015 especially in those younger than 50 years among whom migraine is in the leading causes of disability [9, 11]. The WHO considers a day lived with severe migraine as disabling as a day lived with dementia, quadriplegia or acute psychosis and more disabling than blindness, paraplegia, angina or rheumatoid arthritis. Migraine impacts not only the persons suffering from migraine but also the healthcare system and society by incremental consumption of healthcare resources and reduced work productivity [25, 45]. Our study showed that HRU in terms of the number of visits to HCP, ED, general/family practitioner, neurologist, and psychiatrist in a 6-month period were significantly higher among the migraine sample than non-migraine controls. These findings are consistent with previous studies in Europe and the US conducted in the overall migraine population where ED visits, hospitalizations, and medicines are among the major cost drivers, while the presence of certain symptoms and/or comorbidities leads to further increase in direct costs [7, 17, 25]; as the frequency and severity of migraine increased, the HRU and economic impact to the healthcare system also increased. It should be noted that the low neurologist visit frequency in our study (13.8%) were similar to previous European studies [13, 27], indicating the lack of specialist healthcare availability in Europe.

Previous European studies have estimated the total cost of migraine at between €18 and €27 billion [45, 47]; these studies refer to the total migraine population and are based on prevalence-based calculations and extrapolation of rough estimates on HRU and costing across multiple countries [45, 47]. Country-specific cost of illness studies are needed to provide a more granular approach into the cost of migraine, especially in those who suffer from at least 4 migraine days per month and are often eligible for prophylaxis. It has been estimated that 77% to 93% of all costs associated with migraine are indirect and attributed to impaired or lost work productivity [47, 48]. Previous studies have often looked at the overall population with migraine, the majority of whom suffer from less than 4 MHDs, and, therefore, the total costs associated with migraine may be underestimated.

The findings of the current study revealed that migraine is associated with high burden for those suffering from ≥4 MHDs affecting not only the sufferers (health status and HRQoL) but indirectly the society, employers, and healthcare system [7, 20, 21, 25]. Furthermore, the study also reported the overall prescription medication use for 4–7 EM, 8–14 EM, and CM subgroups were 49.1%, 46.9%, and 68.3%, respectively. That means that, 50.9% of 4–7 EM, 53.1% of 8–14 EM, and 31.7% of CM subgroups are not being treated even when suffering with ≥4 MHDs. Patients need to be treated with medications which result in reduction of migraine frequency and thus have a substantial impact on improving HRQoL, increasing work productivity, and reducing both activity impairment and associated HRU. Given that the prevalence of migraine peaks in those aged 30 to 49 years—an age of prime productivity on a personal, social and professional level—it is important to address the high unmet need for those affected.

### Limitations

There are several limitations that should be noted and which are inherent to these type of studies [49, 50]. .All data are patient-reported via an online panel-based sample and therefore certain biases may exist, therefore caution needs to be taken when interpreting the data.

Despite using the method of administration and randomization to achieve representativeness of the study sample to that of the general population, some bias may still be introduced; this may be due to the inherent differences across different countries as well as access restriction for specific segments of population such as elderly, institutionalized, and those with severe comorbidities and disabilities. Certain efforts were employed to minimize this such as by telephone recruitment where internet access may be limited. The survey responses were self-reported, and data could not be independently verified. The survey questions were relatively benign, and the survey was confidential, diminishing the incentive to misrepresent one’s reporting. All analyses were run in aggregate and no individual-level analysis was conducted.

The self-reported nature of the NHWS is associated with potential corresponding biases such as inaccurate recall and false reporting (whether intentional or unintentional). For example, diagnoses of migraine or other comorbidities (eg, those used in the comorbidity index) were self-reported and are not verified by a physician or medical record. However, questions on year of diagnosis and type of diagnosing physician were also asked which minimizes the probability of false reporting. There is an inherent recall bias to some of the questions that require retrospectively to report outcomes such as HRU. However, major events like an emergency room visit or hospitalization are less likely to be subjected to inaccurate recall compared to a visit to the general practitioner. However, the panel does take adequate measures to minimize intentionally false HRU reporting. For instance, limiting ranges for the number of visits so extreme values aren’t possible, checking for respondents speeding through the survey, or using adaptive questioning to reduce the complexity of the questions.

While measured variables were accounted for in matching and regression, there is the possibility of groups differing on unmeasured variables that may have an impact on outcomes. Although we have tried to match the 2 sample groups by using propensity score matching across different variables (age, gender, BMI, and other factors used), variables that were not considered and could have impacted the analysis may still exist.

## Conclusions

The findings of the current study reveal that there is an incremental burden due to migraine on HRQoL (mental, physical, and health status), work productivity (both presenteeism and absenteeism), and the utilization of healthcare resources among those who suffer from migraine ≥4 MHDs in comparison to the matched non-migraine controls in the EU5. Moreover, migraine is undertreated as the patients did not have access to appropriate healthcare, suggesting that effective management and preventive treatments are needed to lessen the frequency and burden of migraine.

## Abbreviations

BMI: Body mass index
CCI: Charlson comorbidity index
DALYs: Disability-adjusted life years
ED: Emergency department
EQ-5D: Euroqol-5D
EU5: France, Germany, Italy, Spain and the UK
GBD: Global Burden of Disease
HCP: Healthcare provider
HRQoL: Health-related quality of life
HRU: Healthcare resource use
IRB: Institutional Review Board
MCS: Mental component summary
MHDs: Monthly headaches days
NHWS: National Health and Wellness Survey
PCS: Physical component summary
SF 36 v2: Short-Form 36-Item Health Survey version 2
WPAI: Work productivity loss and activity impairment
WPAI-GH: Work productivity and activity impairment
YLDs: Years lived with disability
